# The case for BioEffective Surfaces for Transport—a call for interdisciplinary action to further antimicrobial materials in the transport sector

**DOI:** 10.3389/fpubh.2025.1576636

**Published:** 2025-04-09

**Authors:** James Redfern, Jo Flint, Zhenyu Jason Zhang, Dimitrios A. Lamprou

**Affiliations:** ^1^Department of Natural Sciences, Manchester Metropolitan University, Manchester, United Kingdom; ^2^School of Chemical Engineering, University of Birmingham, Birmingham, United Kingdom; ^3^School of Pharmacy, Queen's University Belfast, Belfast, United Kingdom

**Keywords:** antimicrobial materials, antimicrobial coatings, mass transport, frequently touched environmental surfaces, pathogens

Following the emergence of the pandemic virus SARS-CoV-2, concerns relating to spread of microbial pathogens in every-day environments became front and center in the lives of citizens across the globe. This has resulted in a public who are more educated on potential threat of pathogenic microorganisms in the built environment, and directly resulted in almost all institutions and business changing their physical real estate to limit the spread of pathogens (e.g., installation of Perspex screens, one-way systems, and installation of hand sanitizers) (1). With increased awareness of pathogen transmission, and desire for personal safety and hygiene, and changing regulation, alongside the expectation that SARS-CoV-2 will continue to impact on the global population for some years to come, it is sensible and pragmatic to discuss long-term strategies for pathogen control in environments and systems such as mass transport.

During the SARS-CoV-2 pandemic, the mass transport services were a focal point of discussion relating to the potential spread of pathogens notably due to the number of people in close contact with each other, repeatedly touching surfaces that have been touched by a significant number of people since their last physical clean or disinfection. During the financial year 2019/20, there were more than 3.1 billion journeys by rail and 4.5 billion journeys by local bus services in the UK (2). Additionally, UK airports handled 297 million terminal passenger movements in 2019 (2). Globally, there was 4,162 billion kilometers of passenger rail journeys completed in 2019 (3), and over 38 million passenger aircraft flights forecast in 2024 (4). Previous studies have demonstrated the increased risk of pathogen infection correlated with the use of public transport (bus and tram), e.g., in Nottingham (5), along with a correlation between those infected with an influenza-like disease and use of public transport in London (6). When considering bacteria specifically, numerous studies around the globe have demonstrated the presence of potentially pathogenic bacteria on frequently touched surfaces or in the air around them, for example (7) who investigated the London Underground, whilst others investigated train stations (8) inside buses (9) and inside airports (10). Equally, aircraft can harbor potentially pathogenic microorganisms, with one recent review (11) demonstrating frequently touched surfaces such as tray tables, armrests, seat covers, doorknobs and toilet flush buttons hot spots for microbial contamination—noting the inclusion of antimicrobial materials into these products as a potential solution.

In recent years, the increasing risks posed by antimicrobial resistant bacteria (ARB) have highlighted the need for effective control of microorganisms in scenarios where spread of potentially pathogenic microorganisms may be heightened or pose significant concerns and challenges (12). When such microorganisms are deposited on a surface in small numbers but grow rapidly (exponentially) to reach a number capable of causing infection and disease in humans they can have direct consequence on personal health, economic and healthcare burden. If allowed to establish on a surface, these microorganisms can also grow into communities, which are hard to remove or kill, known as a biofilm (13). Such pathogenic microorganisms are generally passive in their movement around an environment. For example, some microorganisms can be spread in aerosol (inside small droplets of water), generated biologically (e.g., breathing or coughing) or mechanically (e.g., air-conditioning systems), and some may transfer from an animated object to another (e.g., human-to-human contact) or from a fomite (an inanimate object harboring a pathogenic microorganism) to an animate object (e.g., human) (14).

Whilst approaches to controlling pathogenic microorganisms on surfaces in transport already exist, such as extensive and increased sanitization (as demonstrated during the SARS-CoV-2 pandemic) and disinfection via UV irradiation, these can be expensive due to the requirement of new machines/equipment, and once the sanitation/disinfection process has concluded, surfaces and touch materials are immediately liable to re-contamination—particularly in such busy, high-throughput locations as transport vehicles and stations. When this important shortcoming of cleaning, sanitation and disinfection is acknowledged, an “always on” solution (that being a solution that is continuously offering antimicrobial effect) such as the implementation of antimicrobial materials would be of huge benefit in combatting the control of pathogens in the public and mass transport systems. Such materials may be innately antimicrobial, produced with a biocide embedded within, or there may be some coatings or treatments that provide antimicrobial properties onto the surface (15). For example, in some hospitals and other end-use environments, copper and silver have been exploited as an antimicrobial material (16). Other materials and additives are now gaining focus in the literature, for example, materials with photocatalytic properties such as certain forms of titanium dioxide, various metal salts and oxides as well as certain dyes [e.g., (17)]. Other metal ions and some organic agents and materials have also been used in nanoform, such as silver, copper and zinc. Additionally, advances in surface design and topography offer another option to reduce the adhesion of pathogenic microorganisms (15). Whilst many options now exist for antimicrobial materials (with more developments and innovations almost certain), understanding potential limitations is also important. For example, how efficacious they are in their intended end-use environment is less well understood (18), meaning that whilst such materials may show potential as an antimicrobial treatment in laboratory setting, how this translates to application is difficult to model. Other materials may require specific conditions to become antimicrobial such as moisture, which may be impacted by local environmental conditions such as temperature or relative humidity, or may only exhibit antimicrobial activity if the microorganism is directly touching the surface. Current approaches to test efficacy of these materials needs further development (19, 20). The global antimicrobial coatings market growing from USD 3.9 billion in 2021, to USD 6.4 billion by 2026 (21); therefore, it is essential that we understand the efficacy of these antimicrobial materials in their intended end-use, to ensure those using, purchasing and installing them can be confident in their decision and expected outcomes. Other concerns related to cost of installation, cost of maintenance, risks of leaching compounds into the environment, cost-effectiveness compared to other interventions (e.g., cleaning) are also all valid. Further to this, for those in research and development of antimicrobial materials to make a solid case for their use, cost-benefit analysis compared to other antimicrobial technologies is essential. As such, these challenges will require cross-disciplinary input to understand and overcome.

Over recent years' the scientific and engineering disciplines within industry and research sectors have continued to innovate and progress the development of antimicrobial materials. However, to progress these developments to a point where they can be implemented as a cost-effective public-health tool, a new truly interdisciplinary and multi-sector approach is required, bringing together engineers, biocide and coating manufacturers, microbiologists, end-users, policy makers among other interested parties. At present, this cross-discipline collaboration is a challenge, holding back the interdisciplinary progress needed to develop and deploy novel and effective antimicrobial materials in the public and mass transport sector. To ensure that these cross-disciplinary boundaries can be bridged, accelerating research and innovation in this area, those involved must engage in meaningful and active dialogue. To that point, the BioEffective Surfaces for Transport (BEST—http://www.thebestnetwork.org) network has launched in the UK. This network, focused on research and innovation in the UK, but with two-way relationships with researchers and industries in other international communities, aims to be a blue-print for other disciplines experiencing the same issues.
